# Submovement interpersonal coupling is associated to audio-motor coordination performance

**DOI:** 10.1038/s41598-024-51629-z

**Published:** 2024-02-26

**Authors:** Julien Laroche, Alice Tomassini, Luciano Fadiga, Alessandro D’Ausilio

**Affiliations:** 1 Center for Translational Neurophysiology of Speech and Communication, Italian Institute of Technology, Ferrara, Italy; 2 Sezione di Fisiologia, Dipartimento di Neuroscienze e Riabilitazione, Università di Ferrara, Ferrara, Italy

**Keywords:** Psychology, Human behaviour, Motor control

## Abstract

Acting in concert with others, a key aspect of our social life, requires behavioral coordination between persons on multiple timescales. When zooming in on the kinematic properties of movements, it appears that small speed fluctuations, called submovements, are embedded within otherwise smooth end-point trajectories. Submovements, by occurring at a faster timescale than that of movements, offer a novel window upon the functional relationship between distinct motor timescales. In this regard, it has previously been shown that when partners visually synchronize their movements, they also coordinate the timing of their submovement by following an alternated pattern. However, it remains unclear whether the mechanisms behind submovement coordination are domain-general or specific to the visual modality, and whether they have relevance for interpersonal coordination also at the scale of whole movements. In a series of solo and dyadic tasks, we show that submovements are also present and coordinated across partners when sensorimotor interactions are mediated by auditory feedback only. Importantly, the accuracy of task-instructed interpersonal coordination at the movement level correlates with the strength of submovement coordination. These results demonstrate that submovement coordination is a potentially fundamental mechanism that participates in interpersonal motor coordination regardless of the sensory domain mediating the interaction.

## Introduction

The situations we encounter in our environment are constantly changing over multiple timescales. The ability to fine-tune the timing of our actions at multiple timescales is thus an essential aspect of our adaptive life. In our social world, the timely coordination of our own motor behavior to that of others is particularly key. Indeed, acting “in concert” with others enables both mundane and remarkable joint performances, from carrying heavy objects together, exchanging speech turns and shaking hands without looking at them, to playing in sport teams or in musical ensembles^1–7^. Because of the growing interest of cognitive science research in joint action, this phenomenon of interpersonal motor coordination (IMC) has received a lot of attention recently^8–13^. IMC has notably been shown to happen spontaneously (i.e., without intention^14^, or despite opposite ones^15^) and across sensory modalities^16,17^. Importantly, IMC too occurs across multiple timescales^18,19^. It has also been emphasized that the sensorimotor mechanisms operating at different timescales work interdependently^20^, but their functional relation and the influence they exert over each other have been poorly investigated. Furthermore, this research domain has explored IMC in the scale of seconds and up, thus targeting the functional chaining of simple movements (i.e., point-to-point movement of a limb) into longer sequences^21^. However, movements are characterized by a rich organization also at faster scales.

**Figure 1:**
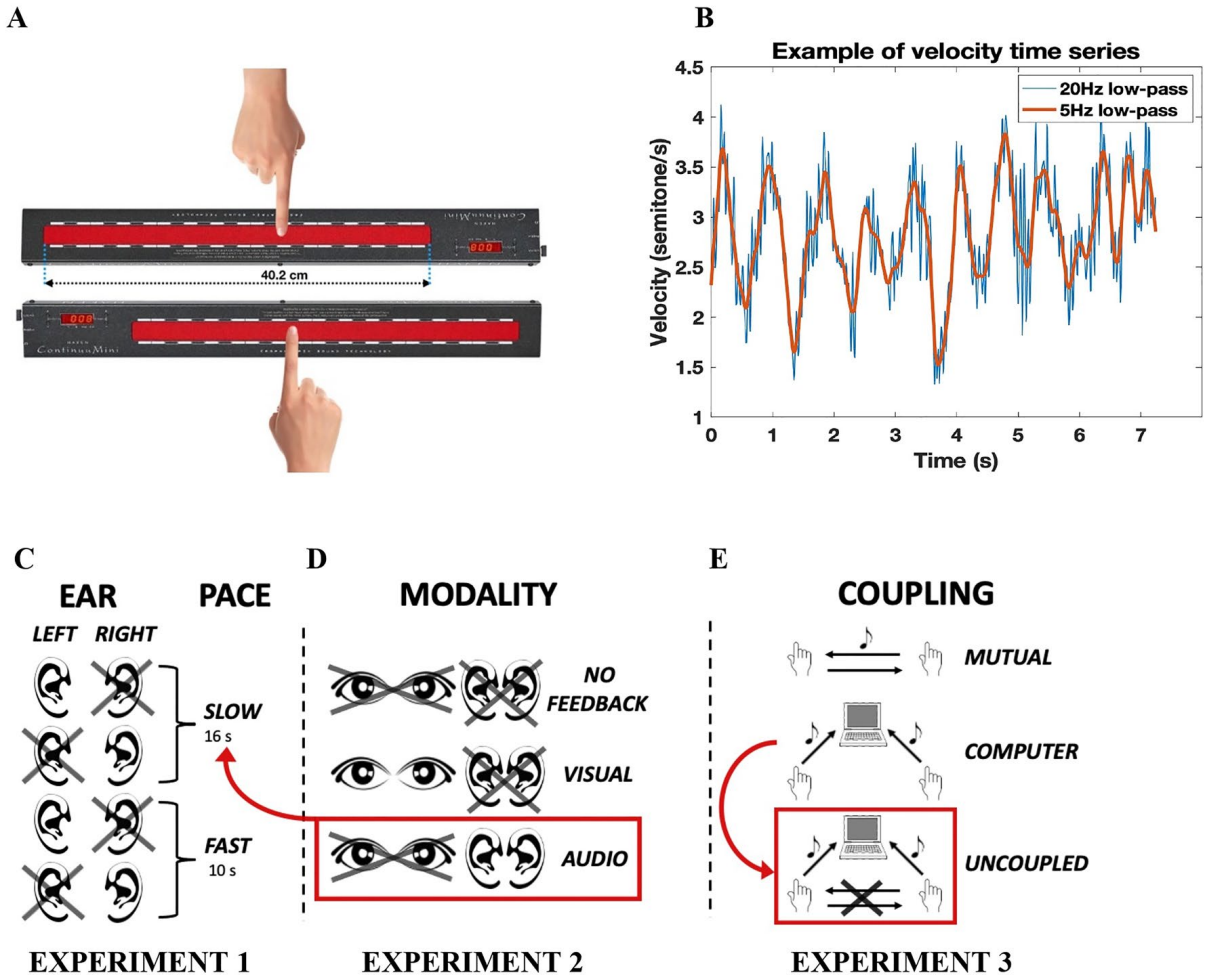
Experimental set-up and velocity fluctuations. (**A**) Participants were comfortably sat so that they could reach the center of the Continuumini bar when their right arm was extended. Participants slid their right index finger over the bar performing slow forearm movements. The bar allowed both the collection of motion data and the synthesis of the feedback tone. One bar was used in experiments 1 and 2, and two bars in Experiment 3. In Experiment 3, partners faced each other, but performed the task eyes closed and could not see each other anyway. (**B**) Example of a portion of velocity time series (one movement taken from Experiment 1), low-pass filtered at 20 and 5 Hz. We can appreciate the periodical fluctuations that mark the clear presence of many submovements (here, 11) along the movement trajectory. (**C**) Summary of the experimental conditions of Experiment 1. Participants were instructed to move at either a SLOW or a FAST pace (making back-and-forth oscillatory movements with periods of 16 and 10 s respectively) and received auditory feedback of their movement either in the LEFT or in the RIGHT ear. (**D**) Summary of the experimental conditions of Experiment 2. Participants were instructed to move at the SLOW pace (16 s). They received either a visual but no auditory feedback (VISUAL condition), or no distal feedback at all (NO-FEEDBACK condition). Since participants of Experiment 2 all took part in Experiment 1, and since the laterality factor did not introduce any difference in Experiment 1, we collapsed the results of the LEFT and RIGHT conditions performed at the SLOW pace obtained in Experiment 1 to compare them with the results of obtained in Experiment 2. We refer to these results as the AUDIO condition in the analyses of Experiment 2 (surrounded by a red rectangle in the figure). (**E**) Summary of the experimental conditions of Experiment 3. Participants received auditory feedback of the mismatch between their position and that of either their partner (MUTUAL) or a synthetic stimulus (COMPUTER). The mismatch between participants’ position and that of their partner during the COMPUTER condition was taken as an UNCOUPLED pseudo-condition (surrounded by a red rectangle in the figure).

One kinematic phenomenon highlighting this fact has been well studied in individual motor control but mostly overlooked in IMC research: submovements^22,23^. Submovements refer to the small and quasi-periodic (about 2–3 Hz) speed pulses that are visible when zooming in on movement kinematic profiles that would otherwise seem fairly smooth (Fig. 1B). These velocity discontinuities mark the boundaries of small segments, or submovements, which are concatenated to form larger trajectories^22–24^. These periodic fluctuations recur in the Delta range^25^: they seem to reflect oscillatory dynamics that are intrinsic to the motor system and that have been identified at the brain level^26,27^. However, submovements are also sensitive to extrinsic factors, such as the introduction of delays in the sensorimotor loop^25^. Submovements might thus reflect the presence of a correction mechanism that is based on sensory feedback^22,28^. In other words, sensing a mismatch between the intended and the actual motor trajectories would trigger the emission of a submovement: a change in the motor output that aims at compensating for that mismatch. As such, submovements are ideal candidates to explain how people use each other’s kinematic cues at the shortest possible scale of motor control to coordinate their whole movement at longer scales^29^. In the interpersonal domain, the presence of submovements has first been observed in dyads of participants who mirrored each other’s improvised oscillatory movements^30^. The amount of velocity fluctuations at the submovement level was not only linked to individual movement properties like speed, but also to interpersonal roles such as leader and follower^31,32^. More recently, it was shown that dyads who had to synchronize finger flexion/extension movements spontaneously alternated the timing of their submovements in a manner that is not (trivially) explained by the concurrent synchronization at the movement level^32,33^. In other words, participants were sensitive to the kinematic cues provided by the (sub)movements of their partner, and they adjusted the timing of their own submovements accordingly.

However, studies on submovements have typically been conducted in the visuomotor domain. It thus remains unclear whether submovements reflect a domain-general mechanism that can be used in solo and dyadic coordination tasks across sensory modalities, or if they only reflect properties that are specific and inherent to visuomotor control. Audiomotor control is a particularly interesting case due to the strong and tight links existing between auditory and motor processes^34–37^. Moreover, auditory feedback (in the form of movement sonification) is known to enhance motor learning^38,39^, for instance by stabilizing trajectories of otherwise visually guided movement^40^, or by increasing the amount of corrective velocity fluctuations during visuomotor tracking^41^. Submovements might thus play a role in audiomotor coordination too. However, the coordination of submovements across persons and even their mere presence in the audiomotor domain have yet to be observed.

In a first experiment we assessed whether submovements were present during audiomotor solo control. We thus asked participants to perform slow forearm movements while they received auditory feedback of their displacement. We then studied the structure of their velocity fluctuations and verified that they are periodically organized in the form of submovements. Then, to evaluate if these submovement properties were specific to the auditory nature of the task, we performed a second experiment in which we tested the same participants in the same task but providing them with either visual feedback of their movement, or no distal feedback at all (proprioceptive feedback only). The results revealed that submovements properties are invariant across modalities of sensory feedback. Finally, in a third experiment, we verified whether submovements were coordinated across partners during a dyadic audiomotor coordination task. Participants had to synchronize their movement with a (computer or human) partner while receiving auditory feedback of their joint performance. When participants are coupled with each other, they spontaneously coordinate submovements according to an alternated pattern. Importantly, the stronger the coordination of submovements, the more accurate partners are at synchronizing at the whole movement level.

## General methodology

In all three experiments participants performed forearm movements to slide their right index finger over a touch-sensitive bar. Motion data was used to sonify movements in real-time. The auditory feedback consisted in a simple sinusoidal sound, the pitch frequency of which was proportionally modulated by finger position in the horizontal axis. The modulation of pitch frequency was chosen because it efficiently conveys human kinematic information^42^. In all three experiments, participants were prompted to move at a slow, yet regular pace.

In all three experiments, we used the Haken Continuumini, a touch-sensitive bar that senses pressure with high spatial resolution (below millimeter) and temporal precision (below millisecond). The bar integrates an internal DSP that uses incoming pressure data to synthesize audio signals in real-time. The bar is 40.2 cm long and 2 cm wide, limiting movement mainly to the horizontal axis (Fig. 1A). We used the (horizontal) position data to determine, in real-time, the pitch frequency of a sinusoidal sound. The mapping between position and pitch frequency was programmed in the Eagan Matrix software, which controls the internal DSP (Digital Sound Processing) of the bar. The auditory output was directly delivered from the bar to headphones (Audio-Technica ATH M70x) via a mini-jack output.

The DSP software allowed us to convert position on the horizontal axis into MIDI notes and MIDI pitchbend data, which represent semitones and fractions of semitones respectively. MIDI data were transferred in real-time and collected along their timestamps via USB with the MATLAB Audio Toolbox (Mathworks). MIDI notes and pitchbend data were then combined in a unique scale, expressed in pitchbend units, where 0 represents the minimal position on the bar toward the left, and 6485 represents the maximal position on the bar toward the right, 28.5 semitones higher. Position data were then converted in semitones (with up to 2 decimals). Timestamped data were interpolated to form evenly spaced time series with a sampling frequency of 100 Hz. The time series of each trial of each participant was then cut into “movements”, which we defined as the interval between the onset at one end of the bar and the arrival at the other end. In experiments 1 and 2, where continuous movements were performed from left to right and vice versa, we used the minimal and maximal positions participants reached during each of their movement to determine onsets and arrivals. In Experiment 3, where participants performed discrete movements from left to right, we used a velocity threshold (0.25 semitone / second) to detect movement onsets and arrivals. The time series of each movement was visually checked to detect and correct potential errors (e.g., false starts). The position time series of each movement were filtered with a two-pass second-order Butterworth lowpass filter and a cut-off frequency of 20 Hz. Filtered position time series were then differentiated to obtain velocity time series. Because movements went into either direction on the horizontal axis in Experiment 1 and 2, velocity values of the movements going from right to left were flipped around 0, allowing us to have comparable signed values for both directions of movement.

To assess how velocity evolved over time, velocity profiles were averaged across movements, trials, and participants in each condition. Resulting profiles showed that movement consisted in an initial transient phase of acceleration, a final transient phase of deceleration, and a long phase with rather stationary velocity in between (see supplementary information). We subsequently excluded the transient parts of the movement (the first second and the last 500 ms) during the analysis of all 3 experiments. This allowed us to focus on the submovements that were observable during the stationary phase.

In all 3 experiments, power spectra were computed for each movement (after the removal of transient parts). We performed band-pass filtering using a two-pass Butterworth filter (3rd order for each single pass) on each velocity time series, applying a sliding window along the frequency axis from 0.3 to 10 Hz in 66 logarithmically (log10) spaced steps and bandwidths (spacing ranging from 0.0335 to 1.4185 Hz). Using the FieldTrip Toolbox, we then processed the resulting time series by Hilbert Transform to study how the frequency composition of the signal evolvds over time. Taking the absolute values of the resulting complex matrices allowed us to obtain frequency-resolved instantaneous amplitude. This step simply allowed used to visually check that power observed at the submovement level was present all along the movements, and not solely at specific points of the trajectory. Then, we averaged the obtained spectra over time points, movements, and trials. This gave us an average power spectrum for each participant in each condition.

In all experiments, the bar was laid on a small 80 cm high table. Participants were comfortably sat on a chair so that (1) moving their right arm horizontally would allow them to comfortably cover the whole range of the bar when pressing it with their right index finger, (2) extending their right arm in front of them would point at the center of the bar. Participants were all volunteers and were paid (12.5 euros) for their participation. All participants were naïve to the purpose of the study, except one author (A. T.) in Experiment 1 and 2. All participants were right-handed (by self-report) and had normal or corrected-to-normal vision. The study and experimental procedures were approved by the local ethics committee (Comitato Etico di Area Vasta Emilia Centro, approval number: EM255-2020_UniFe/170592_EM Estensione). Participants provided written, informed consent after explanation of the task and experimental procedures, in accordance with the guidelines of the local ethics committee and the Declaration of Helsinki. Experiments 1 and 3, including the filling of administrative forms, lasted about 1 h. Experiment 2 lasted about 20 min.

## Experiment 1

### Method

The goal of Experiment 1 was to assess the presence of submovements in audiomotor control. We suppressed participants’ visual feedback by having them close their eyes and provided them with auditory feedback of their movement instead. Participants were asked to perform slow movements at an instructed pace by sliding their right index finger over the bar back and forth (i.e., from left to right and right to left, without interruption). Before each trial, an auditory template was provided. The template simulated the pitch frequency modulation that a movement would produce when performed at constant velocity, over a similar range of positions (i.e., the whole bar from left to right and back, corresponding to the 320–1610 Hz range). The role of this template was to reduce the variability of the paces at which participants moved. The task for the participants was thus to reproduce from memory as accurately as possible the auditory template they just heard by sliding their finger on the bar.

Two within-participants factors were manipulated (Fig. 1C). First, two different movement paces were instructed by two different auditory templates (PACE factor). Participants had to perform movements at 0.0625 Hz (i.e., 16 s per movement cycle which consisted in going back and forth over the bar, 8 s by movement in each direction) in the SLOW condition, and at 0.1 Hz (i.e., 10 s per cycle, or 5 s each movement) in the FAST condition. The PACE factor allowed us to verify if submovement periodicity scaled with the duration of instructed movements they were nested in. Second, the auditory feedback was presented to either the left or the right ear of the headphones (LATERALITY factor, LEFT and RIGHT conditions). This allowed us to verify if inter-hemispheric differences, known to play a role in auditory processing, and particularly in pitch processing^43^, had consequences over task performance.

### Participants

22 participants (14 women, 8 men, aged from 19 to 38 years old; mean 25.10 ± 4.96) participated in this experiment.

### Procedure

Participants were first proposed to freely slide their finger over the bar for half a minute, in order to accommodate with the bar and to experience the auditory feedback of their movement. Next, they were explained that they would perform continuous movements over the bar at a regular pace, and that their goal would be to reproduce, through their movements, the pitch frequency changes they would hear in a prior example (i.e., the auditory template). To make participants familiarizing with the task, they first listened to one cycle of the example, and then performed one cycle on their own immediately after. This procedure was repeated thrice. Then, they listened to the example once more, after which they had to perform uninterrupted movements for one minute. Feedback to correct the behavior were provided only if participants failed at understanding the instructions.

Eight experimental trials were then presented in two separate blocks. Each block corresponded to one of the two PACE conditions. This avoided confusing participants with pace changes. The order between the two blocks was randomized across participants, and the pace of the first block was used during the initial familiarization procedure. A 2-min break was proposed in between blocks, and the familiarization procedure was proposed again, immediately after the break. This time, the familiarization procedure was conducted at the pace at which participants had to move during the second block.

Each block contained two sub-blocks, each consisting in one trial in the LEFT condition, and one in the RIGHT condition. Within each sub-block, the order of presentation of the two LATERALITY condition trials was randomized across participants, with the constraint that both possible orders would be equally experienced by each participant in each PACE condition. Each of the two PACE blocks (SLOW and FAST) thus featured four trials: two in each of the two LATERALITY conditions (LEFT and RIGHT) in each block. Each trial consisted of continuous movements for 2 min, plus the time needed to complete the last cycle of movements they were engaged in at the time of the end of the trial.

### Analyses

We first examined the pace at which participants moved by computing average movement duration (i.e., the time interval between each movement onset and arrival) in each condition. We then examined the periodicity at which velocity was fluctuating by computing the power spectrum of each movement. Next, we extracted the peak frequency of the average power spectra obtained for each participant in each condition. We then verified if the periodicity of submovements was related to the underlying pace of the instructed movement by computing, in each condition, the correlation (across-subjects) between peak frequency and movement duration. Two-way repeated measures ANOVAs were used to evaluate the effects of the PACE and LATERALITY factors on movement duration and on peak frequency. One-sample t-tests were performed separately for each condition (Bonferroni-corrected for multiple tests) to evaluate movement duration against the duration of the template associated to each condition.

### Results

There was a significant main effect of the PACE factor on movement duration (F(1,21) = 830.07, p = 2.0192e-09), but no main effect of the LATERALITY factor (F(1,21) = 2.5521, p = 0.13) nor an interaction between PACE and LATERALITY (F(1,21) = 0.1516, p = 0.70). Average movement duration was significantly higher in the SLOW condition (8.09 ± 1.14 s) than in the FAST condition (5.40 ± 1.38 s;). No significant difference was found between average movement duration and the duration of the template in any of the conditions (SLOW-LEFT: 8.05 ± 1.14 s; t = 0.2166, p = 0.8306; SLOW-RIGHT: 8.12 ± 1.27 s; t = 0.4570, p = 0.6524; FAST-LEFT: 5.32 ± 1.46 s; t = 1.1086, p = 0.3200; FAST-RIGHT: 5.49 ± 1.39 s; t = 1.6655, p = 0.1107). Variability between participants was substantial, but, on average, they moved at the paces they were instructed to.

**Figure 2:**
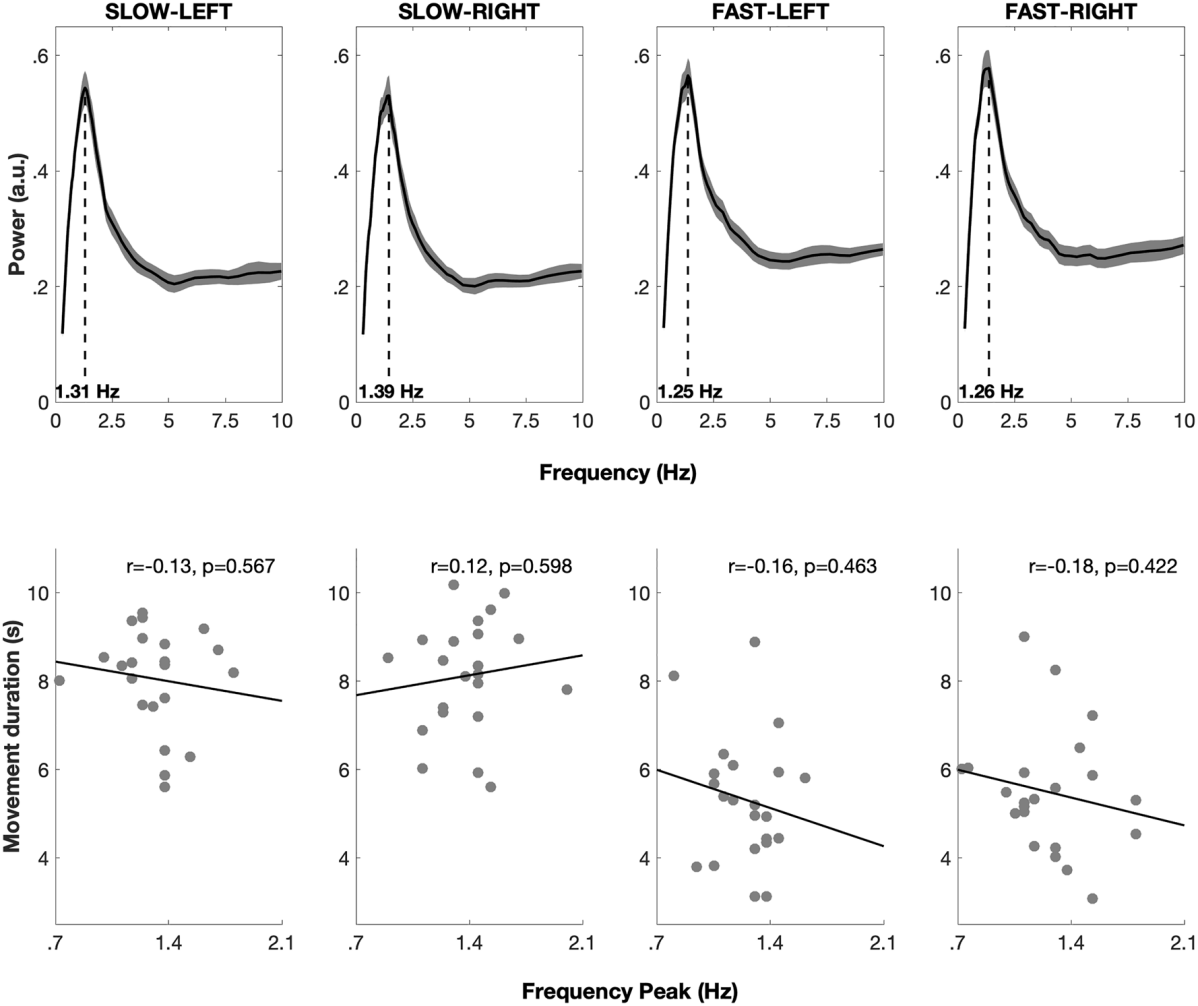
Average power spectra and correlation between individual peak frequencies and movement duration in Experiment 1. (**A**) Average power spectra in all 4 conditions (shaded areas represent standard error and dashed lines indicate the average peak frequency). Average power spectra featured a unique component in all conditions, peaking between 1.3 and 1.5 Hz. (**B**) Scatterplot and correlation between individual peak frequencies and movement duration in all 4 conditions.

Power spectra had similar shapes across conditions, with one main spectral peak ranging from 1.31 to 1.45 Hz (Fig. 2). There was a significant main effect of the PACE factor on the frequency at which peaks were observed (F(1,21) = 4.83; p = 0.04), but no main effect of the LATERALITY factor (F(1,21) = 1.5642; p = 0.22) nor of the interaction between PACE and LATERALITY (F(1,21) = 0.58 ; p = 0.58). Peak frequency was significantly higher in the SLOW (1.35 ± 0.21 Hz) than in the FAST pace condition (1.25 ± 0.20 Hz). However, no significant correlation was found between peak frequency and movement duration in any condition (SLOW-LEFT: 1.31 ± 0.23 Hz; r = −0.13, p = 0.57, SLOW-RIGHT: 1.39 ± 0.23 Hz; r = 0.12, p = 0.60, FAST-LEFT: 1.25 ± 0.19 Hz; r = −0.16, p = 0.46, FAST-RIGHT: 1.26 ± 0.28 Hz; r = −0.18, p = 0.42).

Summing up, participants could easily perform the task at both speeds and regardless of which side we presented the auditory feedback. When zooming in the (stationary part of the) movement, we observed periodic velocity fluctuations typical of submovements (Fig. 1B). Power spectra were similar across all conditions and revealed the presence of a spectral peak between ~ 1.3 and 1.5 Hz. Importantly, peak frequency did not correlate with movement duration, thus suggesting that the periodicity of the submovements is not trivially dependent on the periodicity and duration of the movements. This experiment thus revealed that submovements are present during audiomotor control.

## Experiment 2

### Method

The goal of Experiment 2 was to assess whether the submovements observed in Experiment 1 were specifically linked to the auditory nature of the feedback, or to the presence of a distal sensory feedback at all. As in Experiment 1, participants were asked to perform the same task, but this time in two conditions where the auditory feedback was suppressed (Fig. 1D). In the NO-FEEDBACK condition, participants closed their eyes and moved their finger without receiving any additional distal sensory feedback (i.e., neither visual nor auditory). In the VISUAL condition, they opened their eyes and looked at their finger as it moved on the bar (i.e., visual but not auditory feedback). To provide participants with pace instructions comparable to those we used in Experiment 1, an auditory template was again presented before each trial. As the periodic properties of submovements were essentially unchanged across PACE conditions in Experiment 1, we used only the SLOW condition and panned the presentation of the auditory template dead center (i.e., the signal was identically sent to both LEFT and RIGHT ears).

### Participants

10 participants (6 women, 4 men, aged from 26 to 38 years old; mean 29.30 ± 3.40) participated in this experiment. All of them also participated in Experiment 1.

### Procedure

Instructions and familiarization procedures were the same as in Experiment 1, except for one difference: in each trial, after the presentation of the auditory template, participants performed their first movement cycle with the template playing along. After the first cycle of each trial, the template was muted, and participants continued to perform continuous back and forth movements without any template. This helped them better gauging the adequacy of their movement pace. The experiment then started and was divided in two blocks of two trials. Each block corresponded to one experimental condition (VISUAL or NO-FEEDBACK). The order of presentation of these two blocks was randomized across participants. Each trial lasted as in Experiment 1, i.e., 2 min plus the time needed to complete the cycle participants were engaged in at the end of the trial.

### Analysis

Since the same participants completed both Experiment 1 and 2, data were compared across the SLOW pace condition of Experiment 1 (collapsing the LEFT and RIGHT ear conditions as there was no difference in performance), which here amounts to an AUDIO condition (i.e., auditory feedback only) and the two conditions of Experiment 2 (VISUAL and NO-FEEDBACK). Preprocessing and analyses for Experiment 2 were the same as in Experiment 1. One-way repeated measures ANOVAs were used to evaluate the effects of the condition (NO-FEEDBACK, VISUAL, AUDIO) on average movement duration and peak frequency. Pairwise t-tests Bonferroni-corrected for multiple comparisons were used to compare conditions with each other (i.e., using p-values of 0.0166 as significance thresholds when three comparisons were performed).

### Results

In both the VISUAL and AUDIO conditions, no significant difference was observed between movement duration and the duration of the auditory template (VISUAL: 8.36 ± 1.33 s; t = 0.8497, p = 0.42; AUDIO: 7.87 ± 1.35 s; t = −0.308, p = 0.77). In the NO-FEEDBACK condition, movement duration was significantly longer than the template (9.73 ± 1.68 s; t = 3.2609; p = 0.01). This can be explained by differences observed in the velocity profiles (see Fig. S2). In effect, while the initial part of the movement was performed with comparable velocity changes across conditions, participants gradually decreased their velocity in the course of the movement in the NO-FEEDBACK condition, but they did not do so in the AUDIO, nor in the VISUAL condition. There was a main effect of the condition on movement duration (F(2,9) = 11.889, p = 0.0005). Pairwise post-hoc t-tests revealed that movement duration was longer in the NO-FEEDBACK condition than in the VISUAL condition (t = 4.0618, p = 0.003) and the AUDIO condition (t = 3.8539, p = 0.004), and there was no difference between the latter two (t = 1.3972, p = 0.1958).

**Figure 3:**
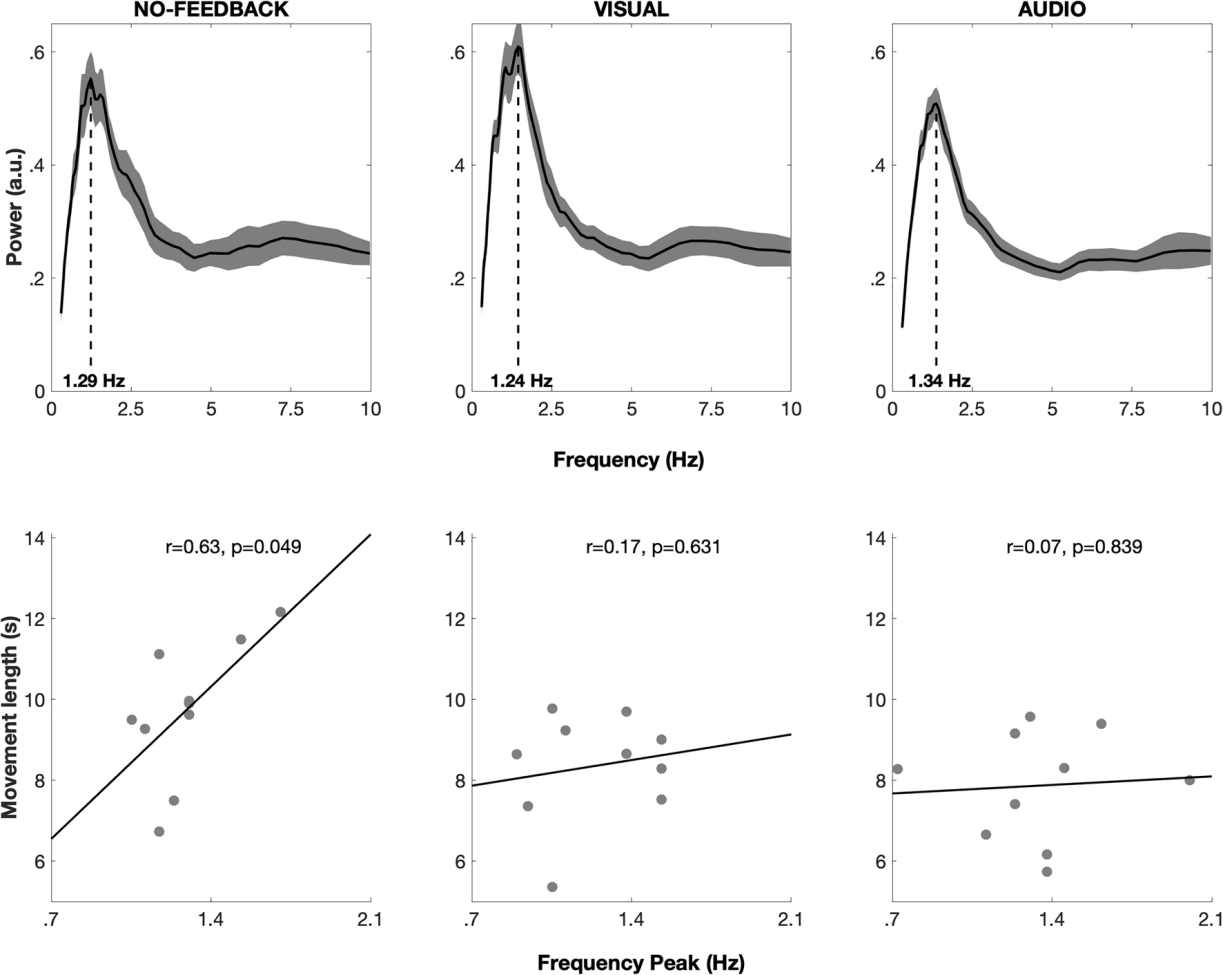
Average power spectra and correlation between individual peak frequencies and movement duration in Experiment 2. (**A**) Average power spectra in all 3 conditions (shaded areas represent standard error and dashed lines indicate the average peak frequency). Average power spectra featured a unique component in all conditions, peaking between 1.2 and 1.5 HZ. (**B**) Scatterplot and correlation between individual peak frequencies and movement duration in all 3 conditions.

Power spectra had similar shapes across conditions, with one main spectral peak ranging from 1.24 to 1.45 Hz (Fig. 3). There was no main effect of the condition on peak frequency (F(2,9) = 0.666, p = 0.5259). There was no significant correlation between movement duration and peak frequencies in the VISUAL condition (r = 0.17, p = 0.63), nor in the AUDIO condition (r = 0.07, p = 0.84), but there was a correlation in the NO-FEEDBACK condition (r = 0.63, p = 0.049), indicating that shorter movement duration (and thus faster paces) corresponded to lower peak frequency (Fig. 3).

Summing up, similar submovement periodicities were observed across all conditions, peaking between 1.2 and 1.5 Hz. Consequently, the periodicity of submovements does not seem to depend on the modality of the sensory feedback, but rather seems to be a pervasive property related to the motor component of the task. In the absence of distal feedback, we also see clear signs of performance worsening at the level of instructed movement properties (i.e., participants did not maintain the instructed pace). Likely, the spared proprioceptive feedback poorly compensates for the precision granted by visual or auditory information in monitoring and maintaining a regular movement pace. Interestingly, only when distal feedback was removed, the periodicity of submovements did correlate with movement time thus suggesting a shift in control strategy. Note, however, that the relatively small sample size in this experiment reduces the statistical power of this correlation analysis, making statistical inference to the population less reliable. Overall, Experiment 2 shows that audiomotor and visuomotor tracking share the same submovement architecture in individual movement control.

## Experiment 3

### Method

The goal of Experiment 3 was to verify if submovements are coordinated between partners when their interaction is mediated by an auditory communication channel, similar to what Tomassini et al. (2022) demonstrated for vision-based interactions^32^. To test our hypothesis, we designed a novel paradigm that provides participants with unique auditory feedback reflecting the combined information of both partners’ movement. More precisely, the tone’s pitch frequency was proportional to the distance between the position of the two partners’ finger on the bar (somewhat mimicking the error signal, i.e., the spatial distance between the two fingers, which is most likely used during vision-based coordination, see^32^). Therefore, the task was to maintain the tone at a target frequency that both partners could hear before starting to move. Importantly, the relationship between the sensory feedback and the adequate motor behavior was univocal. When participants lagged behind their partner, the pitch of their auditory feedback decreased in frequency, indicating that they had to catch up and increase speed. Conversely, when participants were ahead of their partner, the pitch frequency of their auditory feedback increased, indicating that they had to slow down. Ultimately, the task required the partners to coordinate their movements by using feedback from their joint performance.

Two different conditions were designed (Fig. 1E). In the MUTUAL condition, participants had to coordinate their movement with another human partner. In the COMPUTER condition, participants had to coordinate their movement with a simulated movement at a constant speed. This condition had two main functions. First, it allowed us to train participants to move at a particular pace, which they were asked to maintain throughout the entire experiment, including the MUTUAL condition. This helped minimizing the variability of movement paces throughout the experiment and across participants. Moreover, this pace was similar to that required in experiments 1 and 2, increasing the comparability of results between experiments. Second, the COMPUTER condition provided us with a baseline against which to compare interpersonal coordination. Indeed, in this condition, partners performed the same task at the same time, guided by the pace of the same synthetic stimulus. Given that their mutual adaptations were impeded, this provided us with a third, UNCOUPLED pseudo-condition where coordination could be measured between the two non-mutually coupled partners.

### Participants

40 participants (28 women, 12 men, aged from 19 to 35 years old; mean 23.20 ± 3.59) participated in this experiment by forming 20 dyads. One dyad was excluded from the analyses because of the inability of one of the partners to comply with the instructions and produce continuous movements.

### Equipment and stimuli

The equipment was the same as in experiments 1 and 2, except that two Continuumini bars were used (one for each participant in a dyad) and connected to the same computer. Incoming MIDI information related to participants’ fingers position was processed in Usine Hollyhock 5 (BRAINMODULAR). Custom software patches allowed us to compute, in real-time, the relative difference in position between the two partners’ fingers and, therefore, the frequency of the feedback tone for each partner. To account for potential slight position differences at the beginning of the trial between the two partners, the target pitch they had to maintain was determined based on this initial difference; the evolution of this difference throughout the trial determined how the feedback tone deviated from the target pitch. The real-time difference between the position of the participants and their partner was thus computed to estimate the amount of mistuning between them (in fractions of semitones). Then, the frequency of the feedback tone was computed as the sum between a fixed pitch (A3—220 Hz) and twice the amount of mistuning. We used this gain factor to make pitch fluctuations more salient. In the COMPUTER condition, the synthetic stimulus was implemented in a MIDI file that was played in Live 11 (ABLETON), and whose MIDI information was sent to Usine for the computation of the feedback tone. The MIDI file consisted in an 8 s monotonic increase of a pitch frequency from 220 to 1140 Hz. These frequencies corresponded to the pitch frequency resulting from the pressure of the left-end and the right-end position on the bar respectively. The MIDI file thus simulated a partner moving at constant speed from one end of the bar to the other. MIDI information corresponding to the feedback tone received by each participant was sent as MIDI notes and pitchbend via USB from Usine to the Continuumini bars, where the feedback tone was synthesized. We used a very small buffer in Usine to reduce latency to its maximum within the software (about 3.6 ms). Using synchronized audio recordings of finger taps over the bar and of the resulting sound output, we have established that the overall latency of the set-up (i.e., from touch to sound) was near 10 ms. Trigger signals were also played as MIDI files in Ableton Live at the start of each movement. MIDI information of these signals was directly sent to the bars to produce a short, high-frequency (2 kHz) fast rhythmic “alert signal”. These signals instructed participants to get prepared for the start of each movement. They were also sent to MATLAB to synchronize data flows coming from the bars and from Ableton Live.

### Procedure

Participants were recruited in pairs and were given instructions simultaneously. They were presented with how the bars worked and then it was explained that, in this experiment, they would have to slide their finger from left to right and that they would have to find the speed at which the pitch they heard would remain constant in height. They were explicitly told that the pitch would go down if they were too slow, and up if they were too fast. In this experiment, participants only moved from left to right of the bar, instead of performing continuous back-and-forth movements as in experiments 1 and 2.

For familiarization, we used the synthetic stimulus of the COMPUTER condition to start with. This allowed us to progressively set the pace at which we intended participants to move. In these trials, participants were also familiarized with the “alert signal”. This consisted of a short, high-pitched signal. It was first played to indicate when participants should start pressing the left-end of the bar. Then, 1500 ms later, it was played a second time to signal that the trial would begin shortly. The alert signal preceded the onset of the computer stimulus by 1150 ms. Once they had reached the right end of bar, participants were asked to lift off their finger and wait for the next alert signal, and the whole process started again. They repeated this process four times. Next, we familiarized them with the MUTUAL condition. Participants were told that the movement of both would jointly affect the pitch they were hearing, and that, while they had to move at the same pace as in the COMPUTER condition, they would have to do so in a coordinated fashion to succeed in maintaining the target pitch together. As in the COMPUTER condition, the same alert signals were used. They repeated this process four times.

The experiment then started. Participants completed nine trials. Each trial consisted in the above process (place the finger—alert signal—move to the right) repeated for 2 min (8 movement on average). The first trial was always in the COMPUTER condition. It helped us further stabilizing participants’ pace, and it was not included in the analyses. Then, the remaining eight trials were grouped in four blocks of two trials. Each block contained one trial in the COMPUTER condition and one in the MUTUAL condition. The order of presentation of the two trials was randomized across dyads, with the constraint that half of the blocks would start with the MUTUAL condition, and the other half with the COMPUTER condition. Participants were always informed of the condition in which they were about to perform.

### Analysis

We first analyzed movement and submovement properties at the individual level, and then we analyzed their coordination at the interpersonal level. Preprocessing and analyses at the individual level (average movement duration, power spectrum, peak frequency) were the same as in Experiment 1 and 2. Results in the 2 conditions were compared with pair-wise t-tests.

At the interpersonal level, we gauged IMC at the instructed movement level by computing the average amount of mistuning between participants’ and their partners’ position. We did so in both the COMPUTER and the MUTUAL conditions, as well as in the UNCOUPLED pseudo-condition. First, we constructed mistuning time series that reflected how the feedback tone deviated from the target pitch in semitone. Specifically, we computed the continuous difference between the position time series of the two partners, relatively to the initial position mismatch. The mistuning time series were then multiplied by the gain factor (2), to adequately reflect the variations heard by the participants. We removed the initial and final parts where the time series contained movement of only one participant (i.e., the portion that preceded the movement onset of the partner who started the latest, and the portion where one of the partners had already completed the movement). We then computed the absolute mean value of the mistuning time series of each movement, which represented the average deviation from the target pitch, irrespectively of which partner tended to lag behind the other. Results were collapsed across movements and trials. A one-way repeated measures ANOVA was used to evaluate the effects of the condition (UNCOUPLED, COMPUTER, MUTUAL) on average mistuning. Pairwise t-tests Bonferroni-corrected for multiple comparisons were used to compare conditions with each other (i.e., using p-values of 0.0166 as significance thresholds when 3 comparisons were performed).

To evaluate IMC at the submovement level, we performed a windowed cross-correlation (WCC^44^) between partners’ respective velocity time series. As the computer partner was moving at constant velocity, this analysis only made sense in the MUTUAL condition and in the UNCOUPLED pseudo-condition. We filtered the raw position time series at 5 Hz (with a two-pass second-order Butterworth lowpass filter) before differentiating them into velocity time series. We used moving windows of 1 s with a 75% overlap and computed cross-correlation at all lags ranging from −1 to + 1 s. WCC functions were then averaged across movements and trials for each dyad in each condition using Fisher z-transformation, and we used hyperbolic tangent transformation to revert back the results to the scale of correlation coefficients. As we expected velocity time series to be positively correlated with some lag, and negatively correlated at simultaneous times (lag: 0 ms) in the MUTUAL condition^32^, we extracted two indices from the average cross-correlation functions:

1. The average amplitude of the maximum peaks found at both positive and negative lags of the WCC functions in each dyad (MAX-CC). This indicated the extent to which partners’ velocity co-varied, regardless of the lag.
2. The amplitude of the WCC function at lag-0 (Lag-0-CC). This indicated the extent to which the two partners’ velocity underwent opposite changes at simultaneous moments in time.

The resulting indices obtained for each dyad in MUTUAL and UNCOUPLED conditions were then compared with t-tests. Finally, to assess the extent to which submovement coordination played a role in achieving the task goal (maintaining the target pitch), we correlated mistuning performances (at the whole movement level) with the two dyadic indices extracted from the WCC for both UNCOUPLED and MUTUAL conditions.

### Results

Movements were significantly longer in the MUTUAL condition (10.27 ± 1.95 s) than in the COMPUTER condition (9.31 ± 0.87 s; t = −3.399, p = 0.002), and movements were significantly longer in the COMPUTER condition than the computer stimulus itself (8 s; t = 9.299; p < 0.001). As can be seen in the velocity profiles (see Fig. S3), in the COMPUTER condition, participants slowly increased velocity across most of the movement yet barely ever managing to reach the velocity of the synthetic stimulus. From the same plots we can see that while participants moved at an even slower pace in the MUTUAL condition, they did so with a rather stationary velocity across the movement.

**Figure 4:**
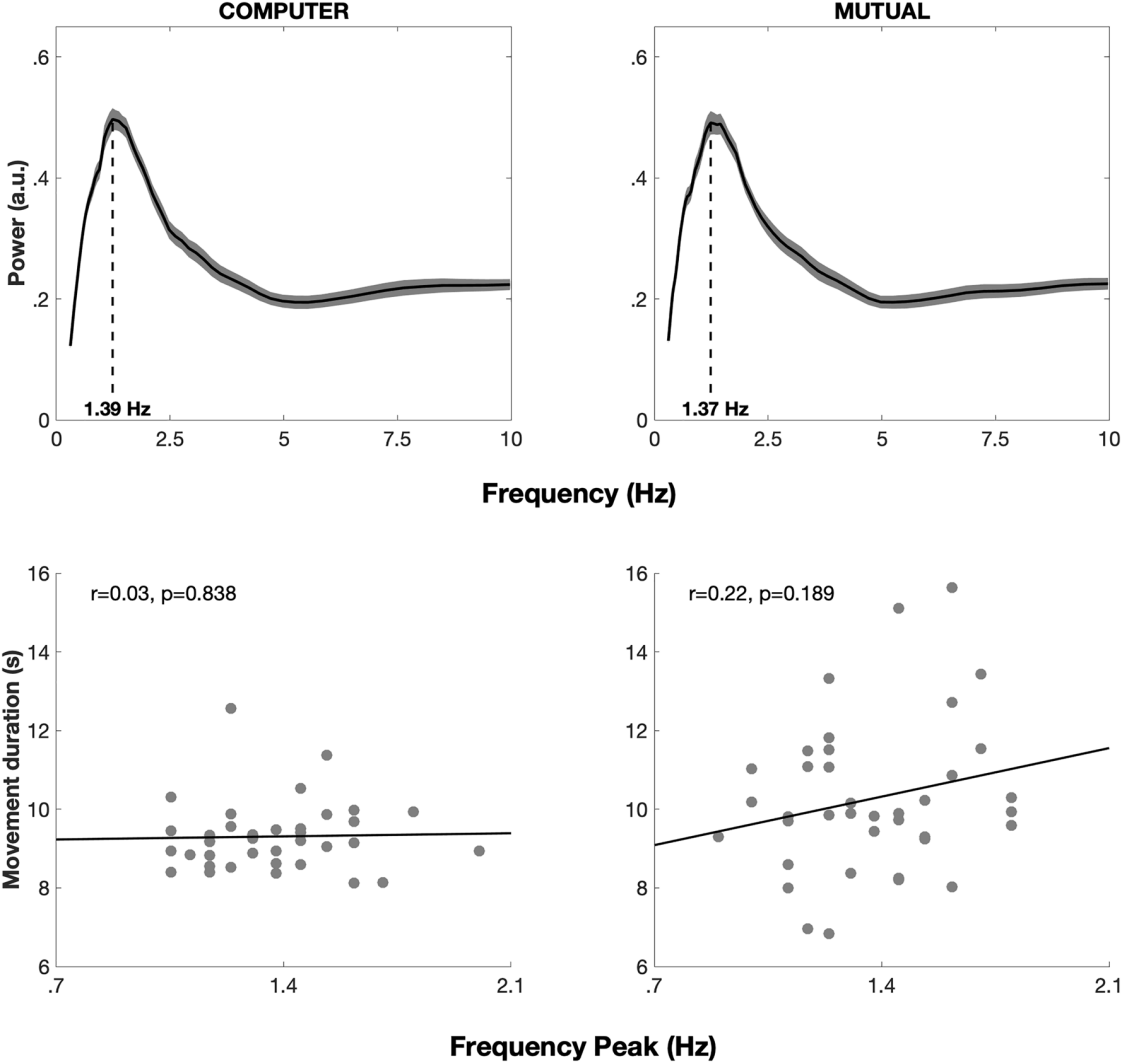
Average power spectra and correlation between individual peak frequencies and movement duration in Experiment 3. (**A**) Average power spectra in both COMPUTER and MUTUAL conditions (shaded areas represent standard error and dashed lines indicate the average peak frequency). Average power spectra featured a unique component, peaking near 1.4 HZ. (**B**) Scatterplot and correlation between individual peak frequencies and movement duration in both COMPUTER and MUTUAL conditions.

Power spectra had similar shapes across conditions, with one main spectral peak at 1.24 Hz (Fig. 4). The frequency at which peaks were observed in the power spectra did not significantly differ across conditions (COMPUTER: 1.39 ± 0.26 Hz; MUTUAL: 1.37 ± 0.24 Hz; t = 0.3848; p = 0.7026). Across participants, correlations between peak frequency and movement duration were significant neither in the COMPUTER condition (r = 0.03, p = 0.838), nor in the MUTUAL condition (r = 0.22, p = 0.189). The periodicities of the submovements were thus not related to the underlying duration of the whole movement.

**Figure 5:**
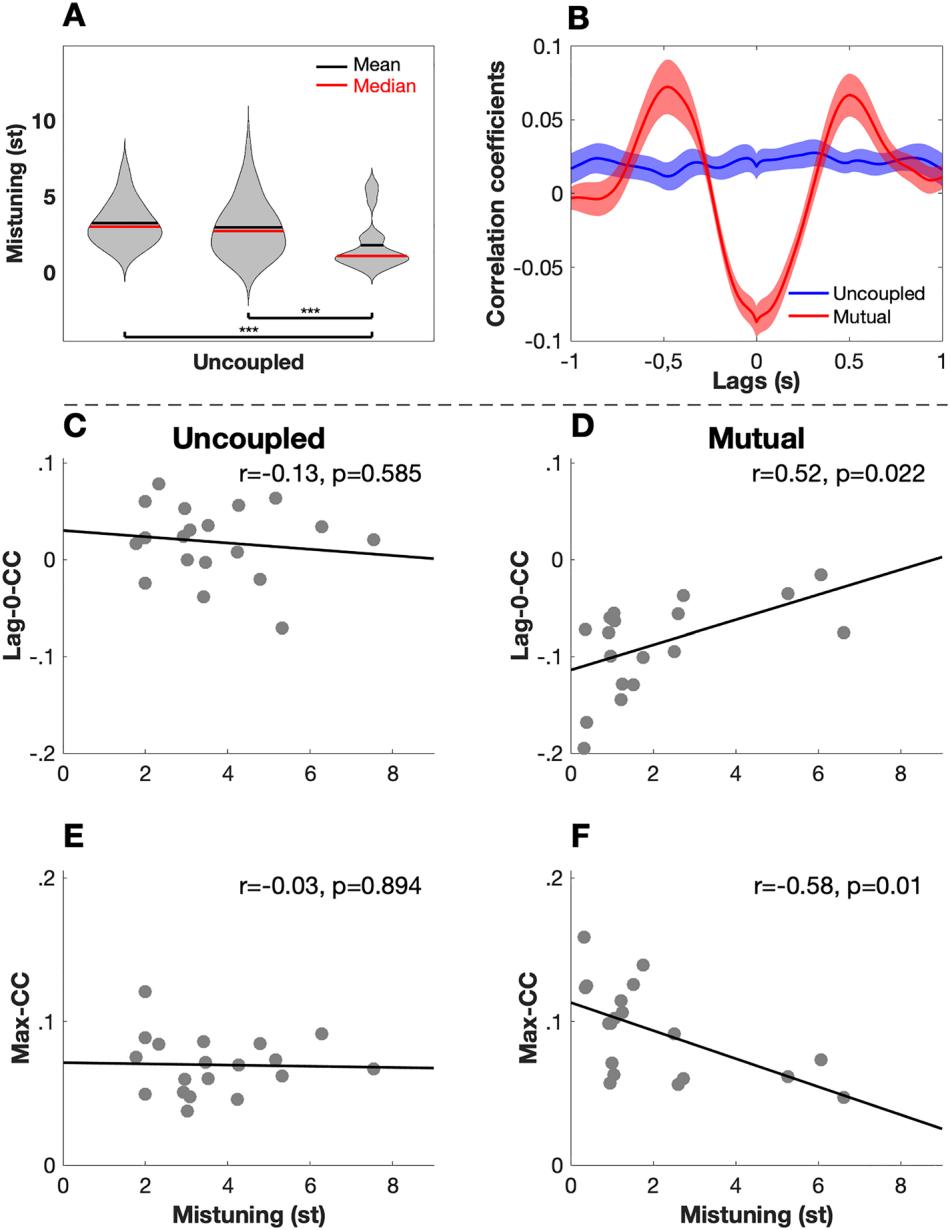
Average mistuning, windowed cross-correlation functions and their relations. **(A)** Average mistuning between participants and their partner (MUTUAL condition and UNCOUPLED pseudo-condition) or the synthetic stimulus (COMPUTER condition). Participants were better able to maintain the pitch frequency of the feedback tone in a smaller range of detuning when they were mutually coupled (***p < 0.025). **(B)** Windowed cross correlations functions between partners (shaded areas represent standard error). In the UNCOUPLED pseudo-condition, no systematic pattern of cross-correlation was observed. In the MUTUAL condition, a systematic pattern characterized by two symmetric positive peaks with lags of about ± 500 ms and a negative peak at lag-0. **(C,D)** Correlation between Lag-0-CC and average mistuning in the UNCOUPLED pseudo-condition (**C**) and the MUTUAL condition (**D**). The correlation was not significant in the UNCOUPLED pseudo-condition it was positive and significant in the MUTUAL condition. **(E–F)** Correlation between Max-CC and average mistuning in the UNCOUPLED pseudo-condition (**E**) and the MUTUAL condition (**F**). The correlation was not significant in the UNCOUPLED pseudo-condition, it was negative and significant in the MUTUAL condition.

Regarding interpersonal performance, there was a significant main effect of the condition on average mistuning (F(2,37) = 12.006, p < 0.0001). Pairwise post-hoc t-tests revealed that the average mistuning was significantly smaller in the MUTUAL condition (1.78 ± 1.65 semitone) than in the COMPUTER condition (2.96 ± 1.80 semitones; t = −3.5912, p < 0.0001), and also in relation to the UNCOUPLED pseudo-condition (3.24 ± 1.36 semitones; t = −4.9535, p < 0.0001; Fig. 5). There was no significant difference between the COMPUTER condition and the UNCOUPLED pseudo-condition (t = −0.8729, p = 0.3884). This reflects the fact that participants were able to use the auditory feedback to co-regulate and coordinate with their partner to ultimately maintain the target pitch within a smaller range of detuning.

The shape of the average WCC functions drastically differed between the MUTUAL condition and the UNCOUPLED pseudo-condition (Fig. 5). In the UNCOUPLED pseudo-condition, the average cross-correlation function between partners’ velocity was rather flat across all lags, with coefficient values close to 0. In the MUTUAL condition, WCC functions featured two symmetric positive peaks, at lags of about (±) 500 ms, and a negative peak at lag 0. Max-CC was significantly higher in the MUTUAL condition (r = 0.09 ± 0.03) than in the UNCOUPLED pseudo-condition (r = 0.07 ± 0.02, t = -2.8042, p = 0.012). Lag-0-CC were significantly lower (i.e., more negative values) in the MUTUAL condition (r = −0.09 ± 0.05) than in the UNCOUPLED pseudo-condition (r = 0.02 ± 0.04, t = 7.4557, p < 0.0001). Thus, compared to uncoupled partners, the velocities of mutually coupled partners tended to show opposite signs (increase/decrease) at simultaneous moments, and similar signs at a lag (of about 500 ms). In absence of information exchange between partners, and despite the presence of a common source of coordination (the synthetic stimulus), partners did not end up producing velocity fluctuations that show any systematic co-variation (beside the common drift in velocity observed in this condition).

To sum up, in Experiment 3, participants performed forearm movements and received auditory feedback of the mismatch between their position and that of the partner. The task of the participants was to maintain the target pitch frequency as best as possible, which could only be achieved by coordinating their movement with that of the partner. The partner was either another human participant or a synthetic and unresponsive stimulus that simulated a movement performed at constant speed. Four main results were observed. First, participants were able to maintain the target pitch within a smaller range of detuning when they were coupled with another human partner than with a synthetic stimulus or compared to the mismatch between their position when they were both coupled with the same synthetic stimulus at the same time. Second, submovements (or velocity fluctuations) recurred with a periodicity that was unaffected by coupling conditions, and which was similar to the periodicity we observed in experiments 1 and 2. Third, participants tended to coordinate their submovements in a systematic, alternated fashion when they were coupled with each other, but not when they were performing the same task at the same time with the same synthetic stimulus. Finally, the degree to which coupled partners alternated their submovements was correlated with their ability to maintain the target pitch at the whole-movement scale.

## Discussion and conclusion

### Summary of the experiments and main findings

IMC is a spontaneous and multimodal phenomenon that can be observed at multiple timescales. A good illustration of the multiscale nature of motor control is provided by submovements (Fig. 1). Submovements consist of periodic speed pulses that become visible when zooming into motor trajectories^22^. Submovements have often been thought to reflect intrinsic dynamics of the motor system^26,27^, and their study has been mostly overlooked by IMC research. Yet, a recent study highlighted that, when two persons synchronized their movement by looking at each other, they spontaneously but systematically coordinated the timing of their submovements with an alternated pattern^32^. The mechanisms underlying submovements are thus sensitive to extrinsic factors such as others’ kinematic cues, and, as such, they involve distal sensory processing^25^. However, submovements have almost exclusively been studied in visuomotor tasks. It is thus unclear if dyadic submovements coordination stems from specific properties of visuomotor control, or if it reflects domain-general mechanisms that operate across sensory modalities.

Here we studied submovements in a series of experiments. In Experiment 1, we first verified that submovements are present in audiomotor solo performance and that they are independent of the movement pace. In Experiment 2, we demonstrated that submovements properties do not change when the modality of sensory feedback changes (i.e., visual and proprioceptive). In Experiment 3, participants had to synchronize their movement with a partner (human or computer) while receiving auditory feedback of the mismatch between their movements (i.e., a measure of synchronization error). Similar submovement properties were again observed and, when they were coupled with each other, human partners also coordinated the timing of their submovements according to an alternated pattern. Moreover, the stronger the coordination at the submovement level, the more synchronized participants were also at the movement level.

To discuss these results together, we broke them down into three main findings: (1) the presence of submovements during audiomotor control and their invariance across sensory modalities, (2) the interpersonal coordination of submovements during audiomotor control, (3) the link between coordination at the submovement level and success in the synchronization task at the movement level.

### Submovement properties are invariant across sensory modalities

When sliding their finger on the bar having a modulated pitch as a feedback signal (Experiment 1), participants produced periodic velocity fluctuations with a peak frequency situated between 1.3 and 1.5 Hz. Importantly, the periodicity of these fluctuations was independent of the speed of their movement. This demonstrates the genuine presence of submovements during audiomotor control. Nevertheless, similar periodic fluctuations were observed in Experiment 2, when the same participants performed the same task with either visual feedback only or no distal feedback at all (proprioceptive only). The same frequency range was also observed in Experiment 3, when participants had to coordinate their movement with a synthetic stimulus or a human partner while receiving auditory feedback of their joint performance. As such, the submovements we observed seemed largely independent of the type of task and sensory feedback, which extends previous data showing their presence even when visual feedback is removed^33,45,46^. In fact, this resonates with the proposition that submovements stem from intrinsic oscillatory dynamics within the motor system^26,27^.

However, the periodicity observed here was substantially slower than what was observed in finger flexion/extension tasks^32^. Actually, the present results are closer to those found in an interpersonal tracking task that involved forearm movements (1.7 Hz^32^). Although these combined observations would suggest that inertia and limbs biomechanics might explain slowing of submovement emission, Noy and colleagues^47^ found even slower submovements in a tracking task (between 0.7 and 1.15 Hz), yet with no difference between arm and finger movements. The contrast between peak frequency variability across studies and the stability of peak frequency across the conditions of our experiments points toward a dependance on the task and context. In our case, even though submovements are poorly affected by haptic feedback^48^, it is likely that the constraints stemming from the friction with the bar played a role in constraining the periodicity of submovements. As such, this speaks against the hypothesis according to which submovements are a pure outcome of the intrinsic dynamics of the motor system or biomechanics. Rather, coherent with the demonstrated importance of extrinsic factors observed in other studies^25^, it seems reasonable to consider the specific periodicity of submovements observed here as emerging from the sensorimotor coupling with the properties of the task environment.

The fact that submovement emission frequency is not coupled to the pace of instructed movements further suggests the presence of a sensory-based error correction strategy: when an error is sensed (e.g., a mismatch when tracking a target), a speed pulse is generated to compensate for it. Interestingly, the only condition where submovements periodicity was correlated with the movement pace was the no-feedback condition in Experiment 2 (but caution must be taken when interpretating this result, given the relatively small sample size in that experiment). In that case, the unavailability of distal sensory feedback imposes corrections to be driven by an internal, proprioceptive, estimation of the error. This could have strengthened the dependence of submovement corrections on the movement pace. In effect, this relationship was such that slower movements saw more frequent re-correction. This phenomenon could stem from the fact that proprioceptive estimation of arm position changes dramatically with movement velocity: slower movements allow for more accurate estimations^49^, which might, in turn, enable more frequent submovement corrections.

In general, these results suggest that submovements reflect intrinsic motor properties that are time-adjusted to compensate for the accumulation of sensory evidence. In line with this we can conceive submovements as the visible trace of an adaptable mechanism that adjusts the rhythmicity with which sensory data is processed to correct and optimize sensorimotor performance.

### Audiomotor dyadic coordination occurs also at the submovement level

The adaptive nature of submovements is far more striking in experiment 3. When pairs of participants had to adjust their movement to each other while receiving auditory feedback of their relative distance on the bar, they systematically coordinated the timing of their submovements with each other. More precisely, participants alternated their emission. This demonstrates participants’ spontaneous tendency and ability to co-regulate and co-organize the relative timing of their submovements. This pattern of results thus closely replicates, in the audiomotor domain, what has previously been observed in the visuomotor domain^32,33^. More recently, interpersonal influences near the submovement frequency range (2–7 Hz) were also observed in a coordination task involving haptic feedback^50^. While frequency ranges drastically vary across studies, the coordination of kinematic fluctuations at faster timescales (the submovement level) than the timescale at which coordination is instructed (the movement level) persists across tasks and sensory modalities.

Syncopated patterns of interpersonal coordination have too been observed in discrete movement tasks: in dyadic tapping, partners tend to follow each other’s timing fluctuations at a lag of one tap^51^. Mechanisms at play in such tasks are often interpreted in terms of error correction^52^: participants react to the mismatch (asynchrony) between their partner’s tap and their own by adjusting the timing of their next tap (e.g., delaying the tap if they previously tapped too much in advance of their partner). Despite the distinctive modes of timing involved in discrete and continuous tasks^53^, both kinds of movement could be subtended by more general mechanisms: when a mismatch between the timing of the self and the other is sensed (i.e., a deviation from the target pitch in our case), an adaptive correction response is triggered in the form of a velocity change in the opposite direction. The lag of that response, identified at about 500 ms in cross-correlation functions, would thus reflect reaction times ensuing from detection, integration, and reaction to the sensory feedback of the joint performance. This mutual process of error correction would thus allow partners to co-regulate their sensorimotor interaction at the shortest possible scale of motor control^ 29,32^.

### Dyadic submovement temporal architecture correlates with movement coordination performance

Importantly, in addition to the extension to the auditory domain, our study adds a new piece of evidence to the findings made in the visuomotor domain^32,33^. In effect, the degree to which partners alternated their velocity fluctuations at the submovement level was linked to their success in the task at the movement level (i.e., maintaining the pitch frequency of the feedback tone by minimizing their relative distance on the bar throughout their movement). The mutual co-adaptation strategy orchestrated by the dyads thus sacrificed synchrony at a short timescale, by adopting an alternated pattern of velocities instead. Such pattern of alternation necessarily increases local variability of the joint performance (i.e., the feedback tone is less stable at the submovement level). However, this apparently ineffective strategy at one timescale is associated with a stabilization of the feedback tone on the longer timescale, that of instructed movements. Co-regulating movement timing by mutually adapting to the timing of each other’s submovement with a lag could thus subserve the optimization of whole movement synchronization (i.e., maintaining the target pitch frequency throughout the task). As such, this result suggests that dyadic coordination at the submovement level can constitute a building block of IMC, a phenomenon that has been strongly overlooked until recently^32,33^.

### Limitation, conclusion, and future directions

In the experiments reported here, participants were instructed to perform slowly oscillating movements. By making the largest portion of the movement stationary, this method allowed us to zoom into and study the inner structure of motor trajectories. Nevertheless, this methodological choice could raise questions regarding the generalizability of the results. In effect, self-preferred paces of periodic movement are typically a lot faster. In fact, they tend to fall within the frequency range of submovements^51^, and this is precisely why rhythmic coordination tasks employing this range of pace cannot properly capture the submovement-based structure of IMC. However, it is worth mentioning that slow-paced movements constitute a key part of our own motor repertoire. We often use slow movements when we learn a new skill (e.g., playing a music instrument, performing a sport or dance move), or when we need to carry out actions or joint actions that require precision (e.g., sticking two pieces of a broken object, manipulating a dangerous object) or force (e.g., lifting a heavy object). Finally, not only do the results in Experiment 1 show the invariance of submovement properties across paces, but it has also been shown in the visuomotor domain that the principle of interpersonal submovement coordination was holding across a wide range of periodicities, up until the respective paces of oscillation of submovements and instructed movements start to mingle^32^. Therefore, our interpretation of the role of submovement coordination in IMC should generalize to a larger class of movement interactions.

In conclusion, submovement thus seem to constitute a channel of communication that helps fostering interpersonal coordination, where the timing of respective movements is syntactically co-regulated and mutually organized. The systematicity and efficiency of the alternated patterns raises questions regarding the spontaneity of such a mode of coordination: to what degree do participants explicitly perceive their partner’s submovements, and, importantly, to what degree do they hold agency over the timely co-organization of their respective submovements? Along these lines, it has recently been shown that participants spontaneously used the kinematic cues of submovements to produce ostensible signs of communication (to make a partner guess the category of an object when most channels of communication were heavily restricted^54^). There seems to be an implicit knowledge that submovements constitute perceivable, meaningful cues that another person can detect and interpret. Better understanding of the relation between agency at the first-person level and submovement coordination from a third-person perspective is however still to be explored.

### Supplementary Information

Supplementary Figures.

## Data Availability

The raw data supporting the conclusions of this article will be made available by the corresponding author (JL), without undue reservation, to any qualified researcher.
