# Supplementary material for: Submovement interpersonal coupling is associated to audio-motor coordination performance
Source: Sci Rep. 2024 Feb 26;14:4662. doi: 10.1038/s41598-024-51629-z (PMC10897171; doi:10.1038/s41598-024-51629-z)
Supplement: Supplementary file 1 — Supplementary Figures. [file 41598_2024_51629_MOESM1_ESM.pdf]

Supplementary information

A

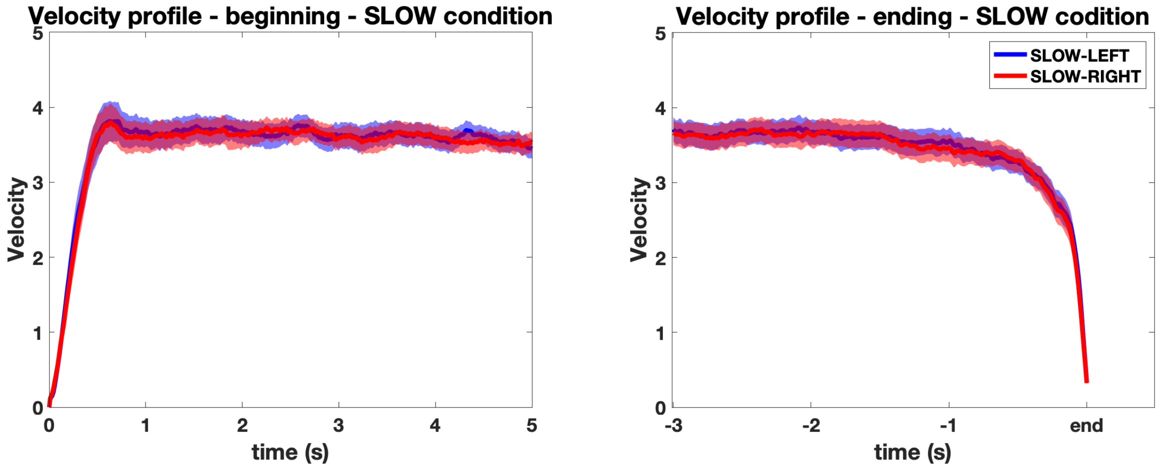

B

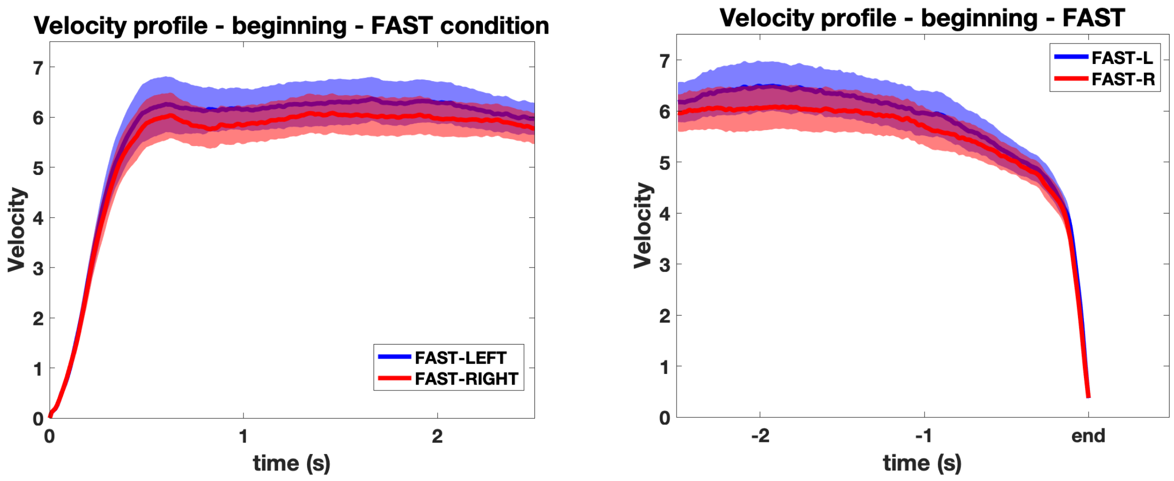

Figure S1. Velocity profiles during experiment 1.
(A) SLOW condition. Movement starts with a steep acceleration curve and a slight overshoot. Then velocity became stationary between 800 and 1000 ms after the movement onset. The end of the movement had a steep deceleration slope, particularly during the last 500 ms.
(B) FAST condition. Movement starts with a steep acceleration curve and a slight overshoot. Then velocity became stationary about 900 ms after the movement onset. The deceleration at the end of the movement was a bit more progressive, with the steepest portion of the curve occurring during the last few hundreds of milliseconds

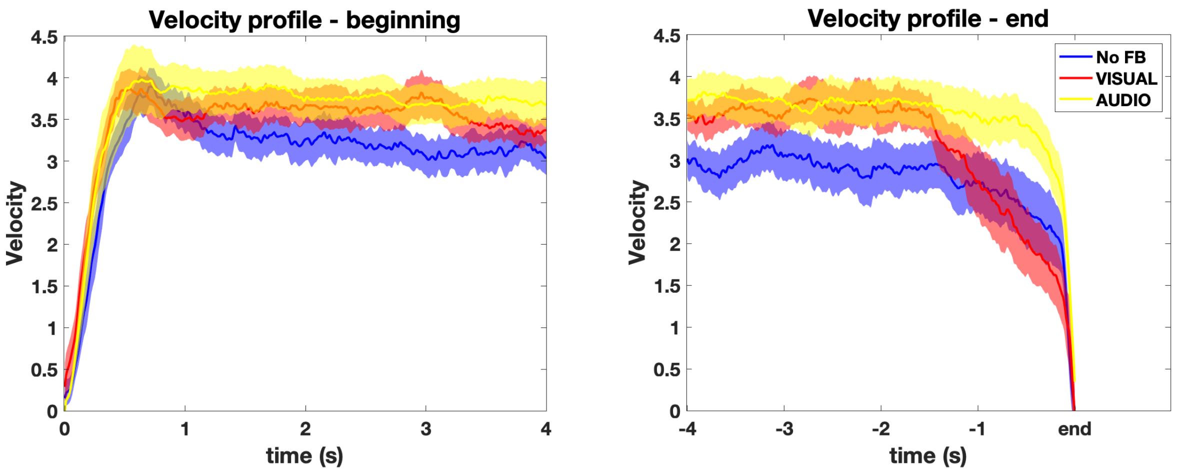

Figure S2. Velocity profiles during experiment 2.
The initial acceleration ramp was similar across sensory feedback modality. In the visual condition, velocity becomes stationary slightly before 1 second, which we also observed in the audio condition. In the no-feedback condition, a slight drift toward lower velocities occurred across the movement, especially after the initial acceleration ramp, but the slope was very slow. The final deceleration part was slightly more progressive in the no-feedback condition than in the audio condition, and much more progressive in the visual condition, starting 1.5 second before the arrival, although the steepest part of the declaration part took place in the last few hundred milliseconds too. Overall, we can appreciate the extent to which the audio feedback helped better maintaining the pace across the movement.

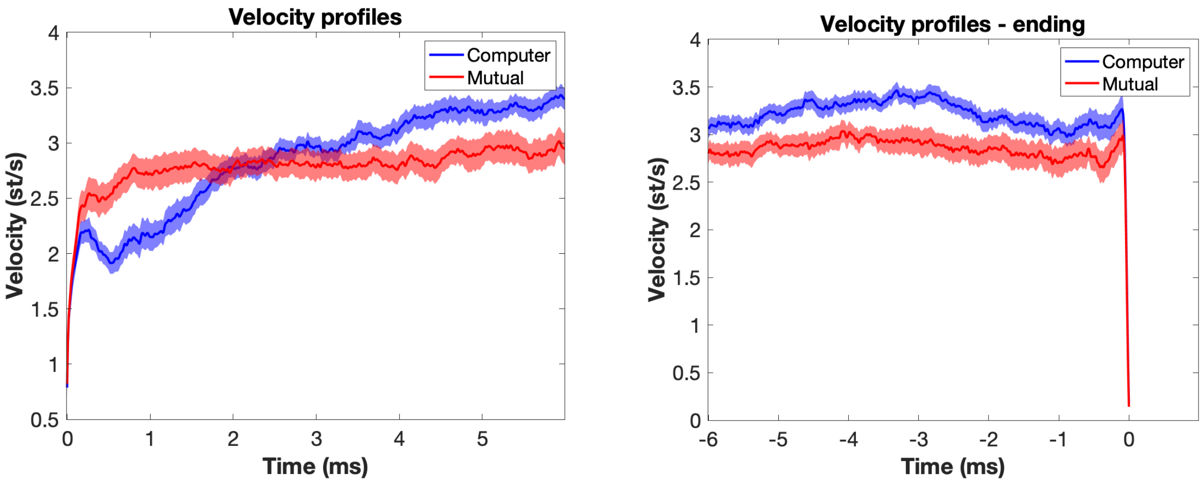

Figure S3. Velocity profiles during experiment 3.
The initial acceleration ramp was different across coupling conditions. In the mutual condition, a steep acceleration initial curve was followed by a more progressive ramp, until it stabilized around a stationary value slightly before 1 second, until the end of the movement which ended abruptly after a short increase. In the computer condition, the initial steep acceleration ramp was followed by a quick velocity decrease. Then, velocity progressively drifted toward higher velocity values across the movement. Velocity slowly decreased again during the last 3 seconds and ended abruptly after a short velocity increase during the last few hundred milliseconds.
